# Detecting Land Subsidence in Shanghai by PS-Networking SAR Interferometry

**DOI:** 10.3390/s8084725

**Published:** 2008-08-19

**Authors:** Guoxiang Liu, Xiaojun Luo, Qiang Chen, Dingfa Huang, Xiaoli Ding

**Affiliations:** 1 Dept. of Surveying Engineering, Southwest Jiaotong University, Chengdu, China; E-mails: rsgxliu@swjtu.edu.cn (Guoxiang Liu); lxj@swjtu.edu.cn (Xiaojun Luo); qchen@swjtu.edu.cn (Qiang Chen); dfhuang@swjtu.edu.cn (Dingfa Huang); 2 Dept. of Land Surveying and Geo-Informatics, The Hong Kong Polytechnic University, Hong Kong, China; E-mail: lsxlding@polyu.edu.hk (Xiaoli Ding)

**Keywords:** permanent scatter, PS networking, radar interferometry, subsidence detection

## Abstract

Existing studies have shown that satellite synthetic aperture radar (SAR) interferometry has two apparent drawbacks, i.e., temporal decorrelation and atmospheric contamination, in the application of deformation mapping. It is however possible to improve deformation analysis by tracking some natural or man-made objects with steady radar reflectivity, i.e., permanent scatterers (PS), in the frame of time series of SAR images acquired over the same area. For detecting land subsidence in Shanghai, China, this paper presents an attempt to explore an approach of PS-neighborhood networking SAR interferometry. With use of 26 ERS-1/2 SAR images acquired 1992 through 2002 over Shanghai, the analysis of subsiding process in time and space is performed on the basis of a strong network which is formed by connecting neighboring PSs according to a distance threshold. The linear and nonlinear subsidence, atmospheric effects as well as topographic errors can be separated effectively in this way. The subsidence velocity field in 10 years over Shanghai is also derived. It was found that the annual subsidence rates in the study area range from -2.1 to -0.6 cm/yr, and the averaged subsidence rate reaches -1.1 cm/yr.

## 1. Introduction

As the largest metropolitan in China, Shanghai is directly close to the sea and Huangpu River. Built on coastal sand and clay that lie 70 meters below the ground surface, this city has been suffering from land subsidence for many years due to overuse of groundwater and rapid construction of skyscrapers [1]. The historical record shows that the most severe subsidence occurred in the 1960s at a rate of over 10 cm/yr - a rate that would have put the city below sea level by 1999 if it had not been slowed down [1-2]. Since then the municipal government has taken some management actions such as pumping water back into ground to mitigate the situation. However, the uneven subsidence at a rate of 1 cm/yr in recent years has still affected or deteriorated facilities such as subway tunnels, buildings, roads, and water and sewage systems, thus resulting in huge economic loss [2].

Monitoring of land subsidence in Shanghai is apparently crucial for predicting potential geological hazards and designing compensation strategies. Over the past decades, the subsidence data has been collected on a regular basis by the conventional methods such as precise leveling and GPS [1-2] which are time consuming, point-based and lack fine details. In recent years, we have focused on exploring a new technique called differential interferometric synthetic aperture radar (DInSAR) to provide another choice for efficiently detecting subsidence in Shanghai [3-4]. It is well known that DInSAR is viable for regional deformation mapping with some prominent advantages such as high sensitivity to motion and fine spatial resolution. Deformation extraction relies on comparison of phase values between SAR images acquired at different time over the same area [5]. However, the full operational capability of DInSAR in deformation monitoring has not been achieved yet. The major sources of uncertainty in interferometric deformation measurements are temporal decorrelation and atmospheric influence [5-7].

To mitigate the aforementioned negative effects, Ferretti *et al.* developed a very generic technique referred to as permanent-scatter (PS) technique to extract deformation information from the multiple interferograms generated with a time series of SAR images [8]. Instead of full-resolution analysis, the PS technique performs modeling and analyzing on PS targets, i.e., hard objects such as buildings, rocks, bridges and dykes, which can maintain steady radar reflectivity even over months to years. On the basis of the basic strategy of PS technique proposed by Ferretti *et al.* [8-9], this paper aims to improve both accuracy and reliability for subsidence detection in Shanghai by considering spatial autocorrelation and parameter adjustment. With the use of multiple interferograms, the analysis of subsiding process in Shanghai is performed on a strong network which is formed by connecting neighboring PS points. Such an approach is thereafter referred to as PS-networking SAR interferometry. Its algorithm validation is conducted using 26 C-band SAR images acquired by the satellites ERS-1 and ERS-2 of the European Space Agency (ESA) from 1992 to 2002 over Shanghai.

This paper is organized as follows. This part is followed by a brief description of data preprocessing and PS-network formation. After this, we present the methodologies of data modeling and parameter estimating. The testing results are then shown and discussed. Conclusions are given in the final section.

## 2. PS detection and PS-network formation

**Figure 1.**
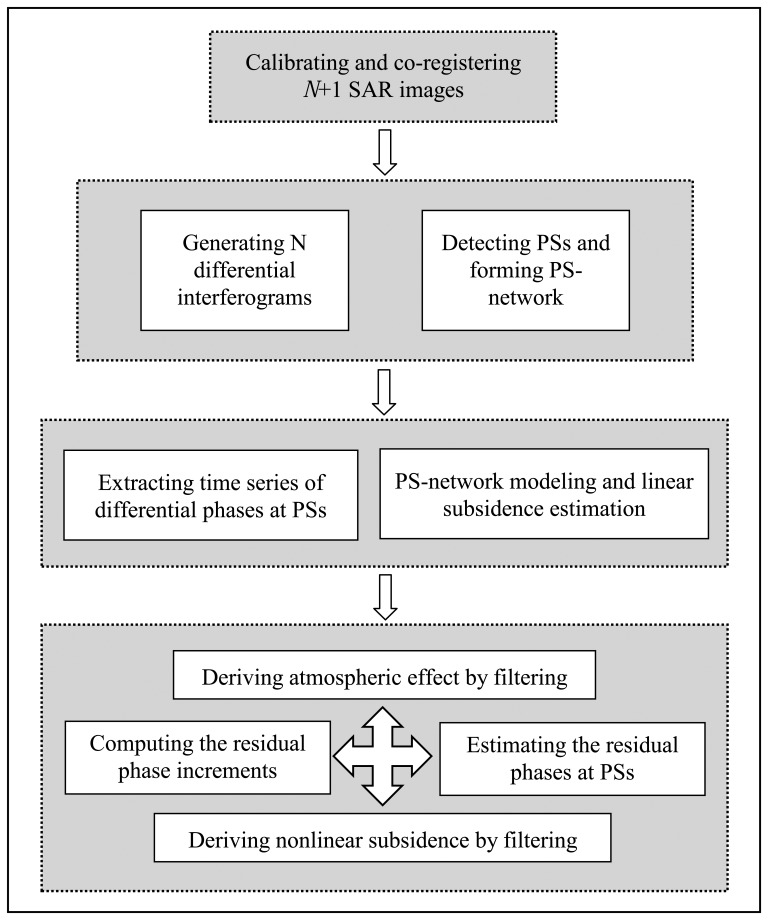
Flowchart of PS-networking SAR interferometry.

Unlike the conventional DInSAR only dealing with a single interferogram, the PS-networking SAR interferometry utilizes the multiple interferograms to isolate deformation information from atmospheric and topographic effects. Figure 1 shows the main procedures of PS-networking SAR interferometry being used for estimating subsidence in Shanghai.

Given *N*+1 SAR images acquired at different time over the same area, they are first ranked by imaging date order. One of them is then selected as the unique master image, while the remaining *N* SAR images are used as the slave images, and thus resulting in *N* interferometric pairs and *N* interferograms.

To guarantee the quality of all the interferograms, we select the optimal master image by maximizing the joint correlation (JC) of all the images with [10]

(1) $$\gamma^{m}=\frac{1}{N}\sum_{k=1}^{N}c\;(B_{⊥}^{k,m},B_{c}\;)c\;(T^{k,m},T_{c}\;)c\;(f_{DC}^{k,m},f_{c}\;)$$

where the function *c* is defined as

(2) $$c\;(x,a\;)=\{\begin{array}{rr} 1-\frac{\left|x\right|}{a} & x<a\\ 0 & x\geq a \end{array}$$

In equation (1), γ*^m^* denotes JC value when image *m* is used as the master; $B_{⊥}^{k,m}$, *T^k, m^* and $f_{DC}^{k,m}$ are the spatial baseline (SB), the temporal baseline (TB) and the Doppler centroid difference (DCD) between image *k* and *m*, respectively; index *c* means the coherence. In equation (2) *a* denotes the critical value of SB, TB or DCD. We set the maximum SB, TB and DCD of all the interferograms as their respective critical values. Let every image be the master and *N*+*1* JC values can be obtained with a trial computation by equation (1). The image corresponding to the maximum JC value is chosen as the optimal master image.

Since the accurate co-registration of SAR imagery is a key prerequisite for any change detection, all the SAR images have to be co-registered into the same space with sub-pixel accuracy [5]. *N* slave SAR images are co-registered on sampling grids of the selected master image by maximizing correlation of amplitude data between SAR acquisitions. As the subsequent PS detection is based on the statistical calculation of SAR data, we calibrate all the SAR amplitude images in a similar way as Lyons & Sandwell [11]. The unique radiometric calibration factor of each image is defined and calculated as a ratio of the amplitude of each image (mean of all pixels) to the mean amplitude of the entire dataset. Each SAR amplitude image is divided by this ratio to make the brightness between images consistent and comparable.

In terms of PS detection, existing study shows that the statistical properties of phase data at any time-coherent pixel can be analyzed by the time series of SAR amplitude data [9]. Although our PS detection basically follows the strategy proposed by Ferretti *et al.* [9], we identify the PS candidates on a pixel-by-pixel basis with use of all the co-registered and calibrated SAR amplitude images. First derived are the overall mean *Ᾱ* and the standard deviation (SD) σ*_A_* of the entire amplitude dataset. At each pixel the time series of the amplitude values is extracted to calculate the mean ā and the SD σ*_a_*. We label a pixel as a PS candidate if the following two criteria are satisfied simultaneously,

(3) $$\{\begin{array}{c} D_{a}=\frac{\sigma_{a}}{\bar{a}}\leq 0.25\\ \bar{a}\geq \bar{A}+2\sigma_{A} \end{array}$$

where *D_a_* is called amplitude dispersion index (ADI) [9]. By the second criteria, the false PSs are more easily removed as the lower amplitude means less temporally coherent. We will eventually judge if the PS candidates are true or false by PS networking based on phase data as discussed in the next section.

After selection of all the PSs, we connect the neighboring PSs to form a network which is similar to a conventional geodetic network like leveling or GPS network. It will be seen that such network can provide a framework for modeling and improving parameter estimation and adjustment. Unlike a triangular irregular network (TIN) as applied by Kampes & Adam [10] and Mora *et al.* [12], we freely link the neighborhood PSs using a given threshold of Euclidian distance. Any two PSs *l* and *p* will be connected only if the following criterion is met,

(4) $$S\;(x_{1},y_{1};x_{p},y_{p}\;)=\sqrt{f_{r}^{2}\cdot {\;(x_{p}-x_{1}\;)}^{2}+f_{a}^{2}\cdot {\;(y_{p}-y_{1}\;)}^{2}}\leq S_{0}$$

where (*x*, *y*) are the pixel coordinates within the image space; *f_r_* and *f_a_* are the scaling factors (converting pixel dimension into geometric distances) in range and azimuth direction, respectively; *S*_0_ is the distance threshold (e.g. 1 km) used to form a PS-PS connection which is thereafter called an arc.

**Figure 2.**
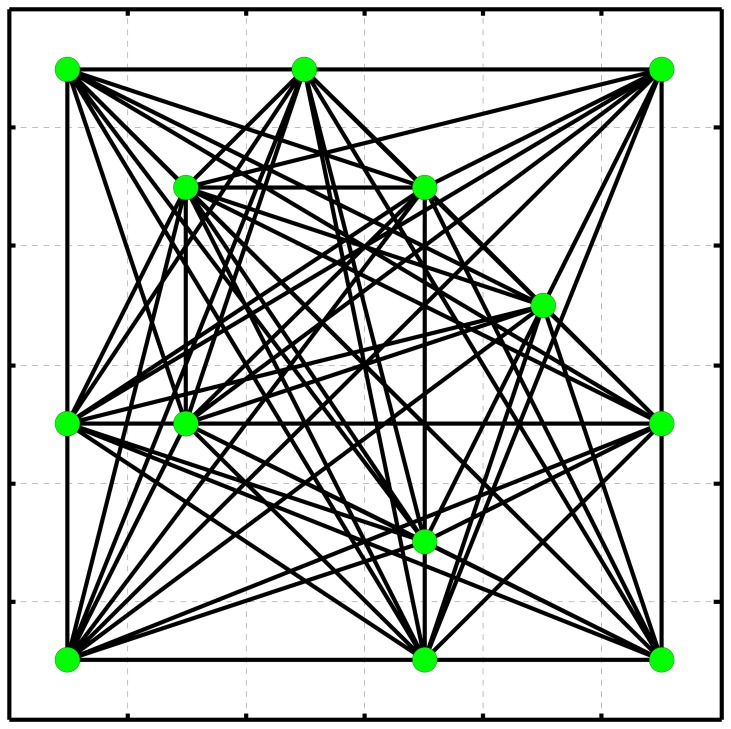
An example of PS network.

It should be pointed out that 0 *S* is generally chosen by mainly considering the atmospheric gradients on the space domain. The faster the spatial variation of atmospheric delay, the shorter the distance threshold. As an example, Figure 2 shows a network, herein termed freely-connected network (FCN), constructed using inequality (4) with several PSs. It should be pointed out that such FCN is much stronger than the TIN in terms of parameter estimation as presented in the next section.

## 3. Spatio-temporal modeling and estimating

### 3.1. Derivation and modeling of differential interferometric phase

Prior to modeling and estimating on the FCN, several procedures must be followed for data reduction. These include computation of the initial interferograms and the differential interferograms. Each initial interferogram can be derived by a pixel-wise conjugate multiplication (equivalent to phase differencing) between the master SAR image and the co-registered slave SAR image. *N* initial interferograms can be obtained in this way. In theory, a direct phase difference at each pixel is due to several contributions, i.e., flat-earth trend, topography, ground motion, atmospheric delay and decorrelation noise [5]. To highlight land subsidence, both the precise orbital data and the external digital elevation model (DEM) can be utilized to remove the flat-earth trend and the topographic effects from each initial interferogram, thus resulting in *N* differential interferograms. It should be emphasized here that no spectral or phase filtering is performed during differential processing in order to avoid deteriorating phase data at PS pixels.

Let us assume that the available DEM has errors and the land subsidence is of linear and nonlinear accumulation in time. The differential interferometric phase at an arbitrary pixel with coordinates (*x*, *y*) from the *i*th interferogram can be modeled as,

(5) $$\Phi_{i}\;(x,y;T_{i}\;)=\frac{4\pi }{\lambda \cdot R\cdot \sin\theta }B_{i}^{⊥}ɛ\;(x,y\;)+\frac{4\pi }{\lambda }\cdot T_{i}v\;(x,y\;)\cos\theta +\phi_{i}^{\text{res}}\;(x,y;T_{i}\;)$$

where $B_{i}^{⊥}$ and *T_i_* are spatial and temporal baseline of the interferometric pair, respectively; λ, *R* and θ are radar wavelength (5.66 cm for ERS), sensor-target distance, and radar incident angle, respectively; ε (*x, y*), *v*(*x, y*) and $\phi_{i}^{\text{res}}\;(x,y,T_{i}\;)$ are elevation error, subsidence velocity, and residual phase, respectively. It should be noted that Φ*_i_* (*x*,*y;T_i_)* is a wrapped phase value within the principal interval of [−π, π). Moreover, the residual phase $\phi_{i}^{\text{res}}\;(x,y,T_{i}\;)$ can be viewed as the sum of several components, including nonlinear subsidence $\phi_{i}^{\text{nlsub}}$, atmospheric delay $\phi_{i}^{\text{atm}}$, and decorrelation noise $\phi_{i}^{\text{noi}}$.

### 3.2. PS-network modeling and linear subsidence estimation

In reality, any regionalized variable follows a fundamental geographic principal; that is the samples that are spatially closer together tend to be more alike than those that are farther apart. The idea of neighborhood differencing is therefore often employed to compensate some spatially correlated errors or offsets. For example, the differential global positioning system (DGPS) may reduce some systematic errors caused by atmospheric delay and orbital uncertainty so that the baseline components (coordinate increments) between two adjacent stations can be determined more accurately. Likewise, the differencing operation along each arc in PS network as shown in Figure 2 is helpful for improving deformation analysis. For the *i*th interferogram, the differential interferometric phase increment along an arc can be derived on the basis of equation (5), such that

(6) $$\begin{array}{l} \Delta \Phi_{i}\;(x_{1},y_{1};x_{p},y_{p};T_{i}\;)\approx \frac{4\pi }{\lambda \cdot \bar{R}\cdot \sin\bar{\theta }}\cdot {\bar{B}}_{i}^{⊥}\cdot \Delta ɛ\;(x_{1},y_{1};x_{p},y_{p}\;)\\ +\frac{4\pi }{\lambda }\cdot T_{i}\cdot \Delta v\;(x_{1},y_{1};x_{p},y_{p}\;)\cdot \cos\bar{\theta }+\Delta \phi_{i}^{\text{res}}\;(x_{1},y_{1};x_{p},y_{p};T_{i}\;) \end{array}$$

where ${\bar{B}}_{i}^{⊥}$, *R̅* and *θ̅* with the obvious symbol meaning are the averaged quantities of two PSs *l* and *p*, i.e., ${\bar{B}}_{i}^{⊥}=\;({B_{i}^{⊥}}_{p}+{B_{i}^{⊥}}_{l}\;)/2$, *R̅* = (*R_p_* + *R_l_*) /2, *θ̅* = *θ_p_* + *θ*. *Δ*ε and Δ*v* are the increment of elevation errors and the increment of linear displacement velocities, respectively. $\Delta_{i}^{\text{res}}$ is the increment of residual phases, which can be extended as

(7) $$\begin{array}{l} \Delta \phi_{i}^{\text{res}}\;(x_{l},y_{l};x_{p},y_{p};T_{i}\;)=\Delta \phi_{i}^{\text{nlsub}}\;(x_{l},y_{l};x_{p},y_{p};T_{i}\;)\\ +\Delta \phi_{i}^{\text{atm}}\;(x_{l},y_{l};x_{p},y_{p};T_{i}\;)+\Delta \phi_{i}^{\text{noi}}\;(x_{l},y_{l};x_{p},y_{p};T_{i}\;) \end{array}$$

where $\Delta_{i}^{\text{nlsub}}$, $\Delta_{i}^{\text{atm}}$ and $\Delta_{i}^{\text{noi}}$ are the increment of nonlinear-subsidence phases, atmospheric phases, and decorrelation noises, respectively.

It should be pointed out that the atmospheric effect and the nonlinear subsidence can most likely be cancelled out by neighborhood differencing embodied in equation (6). It is now readily understandable that we use a short distance thresholding when linking two PSs for networking. The modeling along arcs facilitates the estimation of the two linear increments, i.e., Δε and Δ*v*, which are constant over time.

The theoretical investigation by Ferretti *et al.* indicated that if $\Delta_{i}^{\text{res}}$ is small enough, say $\left|\Delta \phi_{i}^{\text{res}}\right|<\pi$, both Δε and Δ*v* can be indeed derived directly from the *N* wrapped interferograms [8]. In fact, the solution of Δε and Δ*v* can be obtained by maximizing the following objective function [8-9]:

(8) $$\gamma =\left|\frac{1}{N}\sum_{i=1}^{N}\;(\cos\Delta \phi_{i}^{\text{res}}+j\cdot \sin\Delta \phi_{i}^{\text{res}}\right|=\text{maximum}$$

where γ is called the arc's model coherence (MC); $j=\sqrt{-1}$; and $\Delta \phi_{i}^{\text{res}}$ denotes the difference between the measurement and the fitted value, such that

(9) $$\Delta \phi_{i}^{\text{res}}=\Delta \Phi_{i}-\frac{4\pi }{\lambda \cdot \bar{R}\cdot \sin\bar{\theta }}\cdot {\bar{B}}_{i}^{⊥}\cdot \Delta ɛ-\frac{4\pi }{\lambda }\cdot T_{i}\cdot \Delta v.\cos\bar{\theta }$$

Although the objective function is highly nonlinear and the phase datasets are measured in a wrapped version, the two unknowns Δε and Δ*v* of each arc can be determined by searching a predefined solution space (constraint) to maximize the MC value. In the case of perfect phase datasets *g* reaches the best value of 1, while in the case of total decorrelation *g* reaches the worst value of 0. It should be noted that the phase unwrapping can be avoided through the process of function optimization, which is really a challenging task in data processing of the conventional DInSAR.

With equation (8) and (9) we can compute the increments of elevation errors and linear subsidence velocities along all the arcs in the network. By trials with simulated data, we have found that the arcs have an accurate solution for Δ*e* and Δ*v* if γ is not smaller than 0.45. The network is therefore “cleaned up” by deleting some bad arcs and some isolated (false) PS candidates with such MC thresholding. The reduced network can then be treated in a similar way as a leveling or GPS network [13]. The least squares (LS) adjustment procedure is applied to eliminate the geometric inconsistency in the network due primarily to uncertainty in phase data, and thus obtaining the most probable values of the linear subsidence rates and elevation errors at PSs.

Taking the adjustment of a linear-subsidence network as an example, we present some mathematical expressions as follows. A prototype observation equation for an arc is expressed as

(10) $${\hat{V}}_{p}-{\hat{V}}_{1}=\Delta V_{pl}+r_{pl},p\neq 1,\forall p,l=1,2,\ldots ,K$$

where *v̂_l_* and *v̂_p_* denote the linear subsidence rate at PS *p* and *l*, respectively; *r_pl_* is the correction (residual) of Δ*v_pl_. K* is the total number of all the true PSs. Suppose we have *Q* arcs in the network. The matrix form of observation equations can hence be written as

(11) $$\underset{Q\times K}{B}\cdot \underset{K\times 1}{X}=\underset{Q\times 1}{L}+\underset{Q\times 1}{R}$$

where the coefficient matrix *B* is highly sparse and has the nonzero elements of either 1 or -1; *L* and *R* are the vectors for the observations (increments) and the residuals, respectively, of all the arcs; *X* is the vector for the unknown linear subsidence velocities to be estimated at all the true PSs, i.e.,

(12) $$X=[{\hat{v}}_{1},{\hat{v}}_{2},\cdots ,{\hat{v}}_{k}]$$

Furthermore, let the weighting matrix be

(3) $$P=\left[\begin{array}{llll} \gamma_{1}^{2} & 0 & 0 & 0\\ 0 & \gamma_{2}^{2} & 0 & 0\\ \vdots & \vdots & \vdots & \vdots \\ 0 & 0 & 0 & \gamma_{Q}^{2} \end{array}\right]$$

whose diagonal element is the square of MC value previously estimated for each arc. Therefore, the LS solution of the unknowns *X* can be expressed as

(14) $$X={\;(B^{T}PB\;)}^{-1}B^{T}PL$$

The above procedures can also be applied in a similar way onto the elevation-inconsistency network to estimate the elevation errors at all the true PSs. The Kriging interpolator can be used to generate grid data with the results available at sparse PSs [14]. As a remark, we underline that a reference point without motion or elevation error should be selected according to *a priori* information to obtain a unique solution with LS adjustment, and thus making all the estimates be related to the benchmark. Moreover, it should be emphasized that the FCN used here is much stronger in terms of reliability than the TIN. Our simulation study shows that the LS solution derived with the FCN is more accurate than that derived with the TIN even though a small portion of measurements (Δε, Δv) are set as outliers intentionally. This is because the redundancy number in the FCN is significantly larger than that in the TIN.

### 3.3. Extraction of atmospheric effect and nonlinear subsidence

The further analysis focuses on isolating the nonlinear subsidence from the atmospheric delay. For each interferometric pair, the residual phase increment (gradient) at each arc can be first calculated by equation (9). The integration of gradients (i.e., phase unwrapping) of all the arcs in the network is then performed by a weighted least squares method [15], and thus obtaining the residual phases at all the PS pixels for each pair. As seen in equation (5), the residual phase is due to nonlinear subsidence, atmospheric delay and decorrelation noise.

It is possible to separate the nonlinear subsidence from the undesired atmospheric delay because the two terms have different spectral structure in space and time domain [8][12]. In terms of atmospheric perturbation, a high correlation exists in space, but a significantly low correlation presents in time. In terms of nonlinear subsidence, a strong correlation exists in space and a high correlation occurs in time. It is however not easy to discriminate the spectral bands between the nonlinear subsidence and the atmospheric effect if no *a priori* information is available. This implies that an exact separation of the two terms is a challenging task. We basically follow a method by Ferretti *et al.* to isolate nonlinear subsidence from atmospheric delay [8].

The atmospheric phase $\phi_{MI}^{\text{atm}}$ of the master image (common to all the interferometric pairs) can be estimated by

(15) $$\phi_{MI}^{\text{atm}}={\left\{\frac{1}{N}\sum_{i=1}^{N}\phi_{i}^{\text{res}}\right\}}_{LP\_\text{Space}}$$

which means a low-pass (LP) filtering applied onto the mean of the sequence of residual phases. The atmospheric phase $\phi_{SI\_i}^{\text{atm}}$ of the *i*th slave image can then be derived by

(16) $$\phi_{SI\_i}^{\text{atm}}={\left\{\left\{\phi^{\text{res}}\right\}HP\_\text{Time}\right\}}_{LP\_\text{Space}}$$

which means that a high-pass (HP) filtering is first applied onto the time series of residual phases and a LP filtering in space is then applied. The atmospheric phase $\phi_{i}^{\text{atm}}$ of the *i*th differential interferogram is thus obtained as the sum of $\phi_{MI}^{\text{atm}}$ and $\phi_{SI\_i}^{\text{atm}}$. The nonlinear subsidence $S_{i}^{\text{nlsub}}$ contained in the *i*th interferometric pair is finally calculated by

(17) $$S_{i}^{\text{nlsub}}=\frac{\lambda }{4\pi \cos\theta }\;(\phi_{i}^{\text{res}}-\phi_{i}^{\text{atm}}\;)$$

It should be noted that the decorrelation noise can be reduced by the operation of low-pass filtering in space as shown in equation (15) and (16).

## 4. Dataset and subsidence result in Shanghai

To detect subsidence evolution in Shanghai metropolitan (China) by the procedures presented above, we utilize 26 single look complex (SLC) SAR images which are available at hand. They were acquired from 1992 to 2002 by two C-band (wavelength λ = 5.6 cm) radar sensors onboard the satellites ERS-1 and ERS-2, respectively (both operated by European Space Agency). All the images were collected by a nominal radar look angle of about 23° along the descending orbits. With a pixel size of 7.9 m in slant range by 4.0 m in azimuth, each image covers the same area of about 100×100 km whose central location is 121°28′E, 31°10TN. To optimize the interferometric combination, we determined the unique master image by maximizing radar coherence of the entire dataset by equation (1). Eventually the SAR image taken by ERS-2 on May 5, 1998 is chosen as the optimal master image. The remaining 25 images are used as the slave images, thus forming 25 interferometric pairs. Table 1 lists the parameters of all the images, including spatial and temporal baseline with respect to the master image. For detection of PSs, all the 26 amplitude images were calibrated by the procedures as briefed in section 2.

To generate interferograms, all the slave images were co-registered onto the sampling grids of the master image.

**Figure 3.**
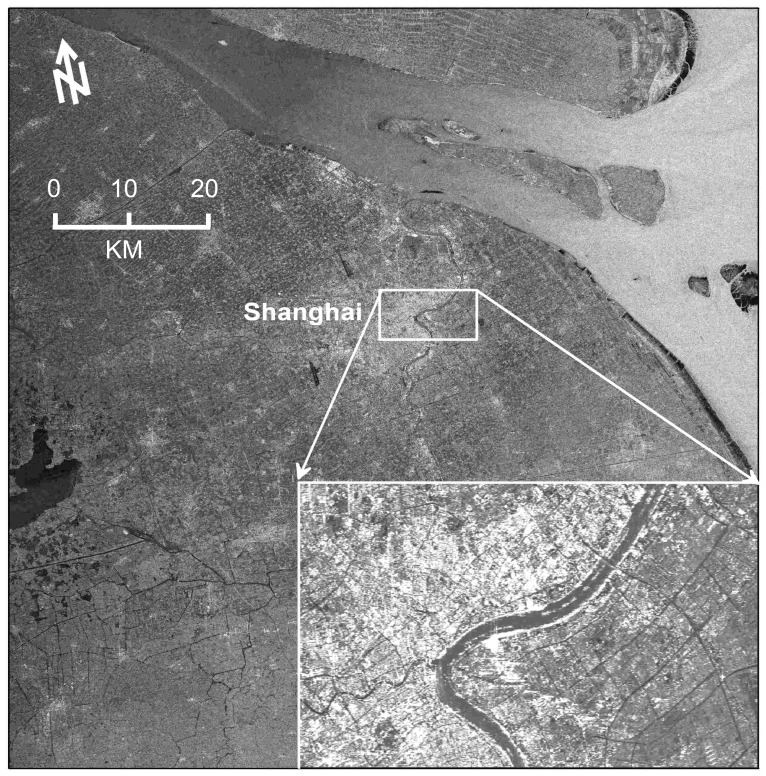
The study area marked by a box onto the master amplitude image.

Existing studies indicate that the most serious subsidence in Shanghai has been taking place around the downtown area, and reached a remarkable value of 2.63 m accumulated from 1921 to 1965 [1-2]. The further data reduction is therefore focused on the main downtown area of 7 km by 12 km. Figure 3 displays the study area of interest (AOI) marked by a box onto the master amplitude image, where the inset shows the enlarged multi-image reflectivity map derived by averaging all the image patches of the AOI. Its radiometric quality has been dramatically improved due to the reduction of speckle noises by averaging. It is clearly visible that Huangpu River wriggles over the study area. The 25 differential interferograms were generated by the “two-pass” method [4-5]. To remove both flat-earth and topographic phases, we use the precise orbit state vectors (about 4-cm accuracy in the radial direction) provided by Delft Institute for Earth-Oriented Space Research in Netherlands [8-10] and a DEM (about 5-m accuracy) which were generated using 1:50000 digital maps provided by State Bureau of Surveying and Mapping, the national mapping agency of China.

**Figure 4.**
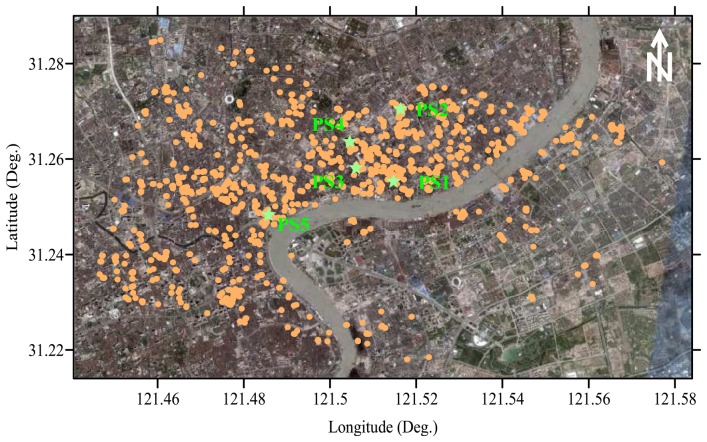
All the detected PSs superimposed onto an optical orthoimage.

The PS candidates were detected on a pixel-by-pixel basis by the statistical computation of time series of amplitude values at each pixel. The pixel is determined as a PS candidate based on the criteria of inequality (3). Figure 4 shows the distribution of all the 1520 PSs obtained in this way, which are superimposed onto an optical orthoimage created with data from IKONOS sensor. In Figure 4, five PSs marked by pentagram and PS1, PS2, …, PS5, respectively, will be used for later analysis of time series of subsidence (see Figure 7). It can be noted that the high density of PSs (about 35/km^2^) appears in the area with dense buildings, while the PSs are rare in some farmlands due to serious temporal decorrelation. We formed a very strong network by freely connecting each PS and all the others if their distance is less than 1 km, as defined in inequality (4), resulting in 4202 arcs.

**Figure 5.**
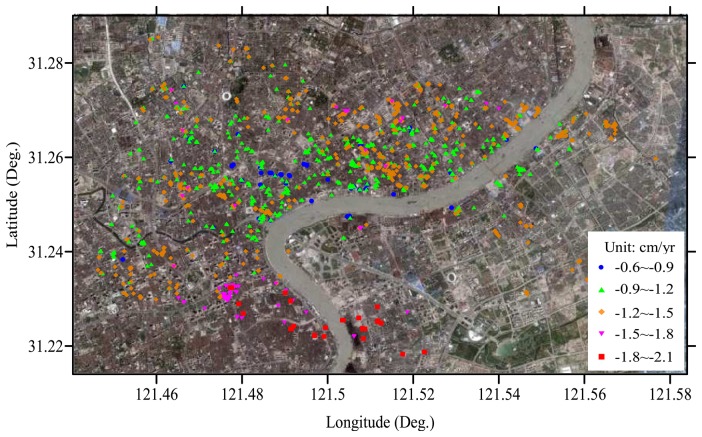
The classed map of linear subsidence rates at all the PSs.

The increments of both linear subsidence velocities and elevation errors between two adjacent PSs of each arc were then estimated by maximizing MC with equation (8). As discussed early, we used a MC threshold of 0.45 to reject low-quality arcs and “bad” PS candidates. 1502 PSs and 4092 arcs thus remained as the valid input of the subsequent LS network adjustment in which PS1 was selected as a reference point for LS solution [3-4]. The linear subsidence rates and elevation errors at 1502 true PSs were derived. Figure 5 shows the classed map of linear subsidence rates (in cm/yr) at all the true PSs. The subsidence rates from 1992 to 2002 in the study area range from -2.1 to -0.6 cm/yr, and the averaged subsidence rate reaches -1.1 cm/yr.

It should be pointed out that the FCN used in our approach is more advantageous than TIN used elsewhere in terms of accuracy and reliability for estimating subsidence rates and elevation errors at PSs, although the former incurs much heavier computation burden than the latter. The reliability with FCN is significantly enhanced because it has much more connections (arcs) between adjacent PSs than TIN. In other words, the total number of redundant observations in FCN is much larger than that in TIN. Hence the LS estimator for FCN is less disturbed by outliers. Our testing results derived with simulated data indicated that the FCN-based LS estimation can efficiently resist against a small portion of outliers in measurements (Δε, Δ*v*). In addition, the FCN tends to remain more PS points than TIN when deleting some “bad” arcs by MC thresholding. The weaker links in TIN may cause more isolated PSs which can not be connected with other PSs, and some true PSs are erroneously rejected. The stronger links in FCN are therefore useful for recovering the finer details of deformation field.

**Figure 6.**
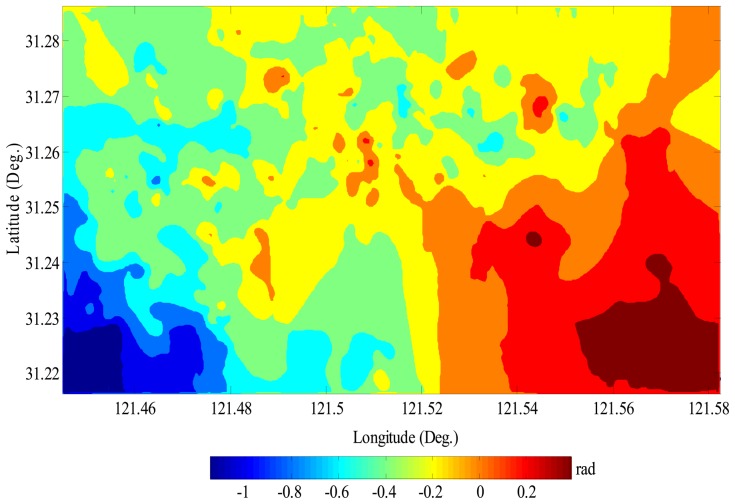
The atmospheric phases in the partial AOI for the master image.

The atmospheric delay and nonlinear subsidence in the study area were finally separated by a time-space filtering method as discussed in section 3.3. Prior to such separation, the residual phases in each differential interferogram were extracted by detrending both linear subsidence and topographic effect. The atmospheric phases of the master image (by ERS-2 on May 5, 1998) were derived by a LP space filtering applied onto the mean of 25 residual-phase images (see equation (15)), while the atmospheric phases of any slave image were estimated by time-space filtering according to equation (16). As an example, Figure 6 shows the atmospheric phases in the partial AOI for the master image, which vary from -1.2 to 0.4 radians, i.e., range change of -5 to 2 mm in radar line of sight. As a remark, we stress that exactly separating nonlinear subsidence from atmospheric artifacts is indeed a challenging task. The further improvement on this point is still required, particularly by integrating *a priori* information on atmosphere and subsidence available from some other monitoring approaches such as GPS permanent tracking network.

**Figure 7.**
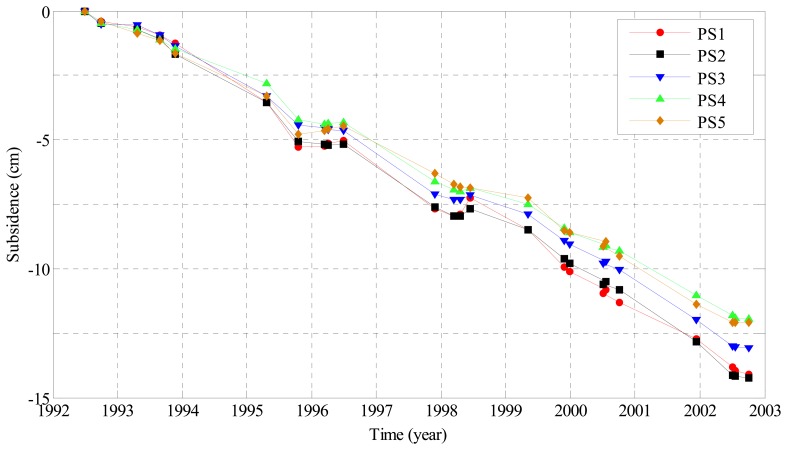
Time series of subsidence at 5 PSs as marked in Figure 4.

**Figure 8.**
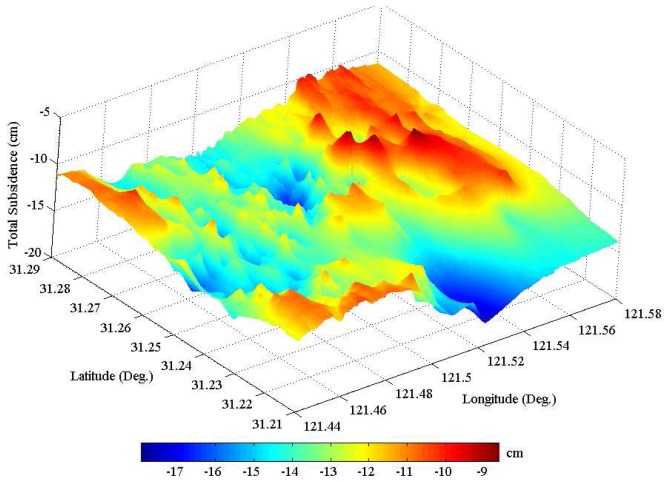
Perspective view of the subsidence field accumulated between June 1992 and August 2002.

After deriving atmospheric phases, equation (17) was used to calculate nonlinear subsidence. The time series of subsidence was eventually obtained as a sum of linear and nonlinear parts. As examples, Figure 7 shows the so-obtained temporal evolution of subsidence at 5 PSs (see Figure 4) in the central part of the study area, where about 15-cm land sinking was accumulated from 1992 to 2002. It is obvious that the linear subsidence trend dominates the nonlinear component with a peak-to-trough variation of about 4 cm in this study area. For visualization, a perspective view of the entire subsidence field is shown in Figure 8, where the remarkable sinking parts can be better appreciated. Maximum and minimum subsidence values are -18 and -9 cm, respectively.

In recent years, both precise leveling and GPS survey have been carried out to monitor subsidence in Shanghai by some authorities [1-2]. Both the first- and second-order leveling are carried out once per year for benchmarks in the downtown area. The annual subsidence rates (see Figure 5) and the accumulated quantity (see Figure 8) estimated with PS-networking SAR interferometry are in good agreement with the leveling subsidence results reported in some open literature [1-2]. This indicates that our approach presented in this study is effective for detecting land subsidence in Shanghai. The current land sinking is highly related to the large-scale urban construction and the overuse of groundwater. Especially from 1992 to 1995, the skyscrapers' constructions are most remarkable [1]. It should be noted that the estimated vertical displacement may also contain the settlement of skyscrapers, and not purely the natural subsidence of the land surface. The annual subsidence rate is however much smaller than that occurring in the 1980's. This is primarily attributed to some mitigation strategies proposed by city managers and planners, which include reducing groundwater withdrawal, increasing river water use, pumping water back into depleted aquifers, and utilizing light materials for construction.

## 5. Conclusions

To mitigate the negative impacts of both temporal decorrelation and atmospheric delay on mapping deformation with conventional DInSAR, this paper has presented an approach called PS-networking SAR interferometry for detection of land subsidence in Shanghai, China. With use of 26 ERS-1/2 SAR images acquired 1992 through 2002 over Shanghai, the time series of land subsidence is analyzed with a very strong network which is formed by freely connecting neighboring PSs according to a given distance threshold. The mathematical models and computing methods are addressed systematically by considering spatial autocorrelation and LS parameter estimation. The linear and nonlinear subsidence, atmospheric effect as well as topographic error were separated effectively in this way. The subsidence velocity field in 10 years over Shanghai was also derived. It was found that the annual subsidence rates in the study area range from -2.1 to -0.6 cm/yr, and the averaged subsidence rate reaches -1.1 cm/yr. The maximum subsidence accumulated in 10 years is up to -18 cm. These are generally in good agreement with the leveling subsidence results reported elsewhere. In addition, the testing results indicated that the FCN proposed in this study is more advantageous than the TIN used elsewhere in terms of reliability for estimating subsidence rates and elevation errors at PSs, although the former incurs much heavier computation burden than the latter.

With further improvement, it is anticipated that PS-networking SAR interferometry would become an operational tool to monitor the slowly-accumulated urban subsidence, and thus complementing the conventional geodetic tools such as GPS and leveling. In China, there are a number of cities which are suffering from land subsidence. Besides Shanghai, the other typical sinking cities include Tianjin and Taiyuan. The reliable and prompt measurements reflecting land subsidence evolution are valuable for assessing and mitigating some geological hazards related to land sinking.

## Figures and Tables

**Table 1.** The parameters of 26 ERS-1/2 SAR images used in this study.

No.	Platform -orbit	Imaging Date	*B*^⊥^(m)	*T* (day)	No.	Platform –orbit	Imaging Date	(m)	*T* (day)
1	E1-04657	1992.06.06	504	−2159	14	E2-14887	1998.02.24	−1239	−70
2	E1-06160	1992.09.19	146	−2054	15	E2-15388	1998.03.31	−487	−35
3	E1-09166	1993.04.17	-36	−1844	16	E2-15889	1998.05.05	0	0
4	E1-10669	1993.07.31	274	−1739	17	E2-20899	1999.04.20	247	350
5	E1-12172	1993.11.13	−639	−1634	18	E2-23905	1999.11.16	−348	560
6	E1-19530	1995.04.10	−207	−1121	19	E2-24406	1999.12.21	−141	595
7	E1-22035	1995.10.02	178	−946	20	E2-26410	2000.05.09	303	735
8	E1-24039	1996.02.19	505	−806	21	E2-26911	2000.06.13	−158	770
9	E1-24540	1996.03.25	−1144	−771	22	E2-28414	2000.09.26	290	875
10	E2-04867	1996.03.26	−1000	−770	23	E2-34426	2001.11.20	−198	1295
11	E1-25542	1996.06.03	−1253	−701	24	E2-37432	2002.06.18	1048	1505
12	E2-05869	1996.06.04	−1104	−700	25	E2-37933	2002.07.23	144	1540
13	E2-13384	1997.11.11	−762	−175	26	E2-38434	2002.08.27	−1021	1575

Note: the B^⊥^ and *T* are the normal baseline and the temporal baseline, respectively.
